# Advances in Rock and Mineral Materials

**DOI:** 10.3390/ma18245576

**Published:** 2025-12-11

**Authors:** Gorazd Žibret, Vilma Ducman, Lea Žibret

**Affiliations:** 1 Department of Mineral Resources and Geochemistry, Geological Survey of Slovenia, Dimičeva Ulica 14, 1000 Ljubljana, Slovenia; 2 Slovenian National Building and Civil Engineering Institute, Dimičeva Ulica 12, 1000 Ljubljana, Slovenia

## 1. Introduction

Earth sciences support society by finding suitable deposits of primary raw materials, while material sciences support production of new and advanced environmental and health-friendly materials that we all use for everyday activities. Modern society is highly dependent on raw materials, which are essential for achieving and maintaining today’s standard of living and a crucial component of the green energy transition. The main source of primary raw materials (PRMs), which are extracted from the earth, is mining activities, while the supply of secondary raw materials (SRMs), which are derived from the recycling of waste materials, is becoming increasingly important [1,2]. To safeguard PRMs, the use of SRMs has been gaining increasing importance. PRMs and SRMs are essential sources for various engineering materials used in the production of everyday consumables and infrastructure. These materials play a crucial role in different sectors, including construction, the food industry, transportation, energy production and supply, telecommunications, household appliances, products for the green energy transition, packaging, etc. [3,4,5,6,7]. Although there is no standard global classification of “mineral materials”, these materials can generally be divided into various groups, including (a) iron and ferro-alloy metals (i.e., Fe, Cr, Co, Mn, Mo, Ni, Nb, Ta, Ti, W, and V); (b) non-ferrous metals (i.e., Al, Cu, Sb, Pb, Zn, REE, semiconductor materials, etc.); (c) precious metals (Au, platinum-group metals, and Ag); (d) industrial minerals (various non-ferrous minerals or mixture of minerals that are used for various industrial processes, like quartz sand, clay, bentonite, diatomite, feldspar, gypsum, phosphate rock, potash, salt, sulfur, talc, etc.); and (e) mineral fuels (coal, lignite, natural gas, petroleum, uranium, etc.). Additionally, “rock materials” are used in construction as aggregates (sand, gravel, and crushed rock). Aggregates are utilized as a basis for infrastructure and construction projects, filler in concrete, and materials for embankments, among other uses. The reprint of this Special Issue “Advances in Rock and Mineral Materials” presents recent advancements in geo- and material science, focusing on studies related to clays, development of new forms of cement and their applications, new advancements in mineralogy and metal recovery, and the mechanical properties of rocks.

In addition to the above-mentioned mineral materials, one of the most essential RMs in construction and other industries is clay. Clay is a fundamental raw material used in the production of fired clay bricks [8]. Additionally, it is also increasingly utilized in the production of unfired clay bricks, rammed earth walls, and other earth-based construction materials [9,10,11,12]. These materials are gaining importance and rely on locally available clay sources. Natural clays are also adsorbent minerals that can effectively cleanse contaminated substances [13,14]. Zeolitic tuffs have been used in construction since prehistoric times, mostly as dimension stones, lightweight aggregates, or additives for the production of cement mixtures [15]. Recently, zeolitic tuffs have become increasingly important for reducing toxic cation and anion concentrations in aqueous solutions in wastewater treatment and soil remediation [16]. One advanced low-energy and low-carbon building material is the belite sulfoaluminate cement clinker, which requires significant amounts of aluminum (Al) sources [17]. The most common natural source of Al is bauxite, which can be replaced by various secondary Al sources [18,19]. This requires detailed mapping and characterization of locally available Al-rich secondary raw materials [20]. Secondary raw materials (SRMs) can play multiple roles in the production of cement and concrete. They can partially replace raw materials used in clinker production, serve as mineral admixtures or aggregates in concrete [21], and act as natural precursors in alkali-activated materials [22,23,24,25,26,27]. Additionally, secondary raw materials are essential for extracting valuable elements [28,29,30]. Such circular economy approaches are crucial for meeting decarbonization targets [31,32].

This Special Issue “Advances in Rock and Mineral Materials” presents eleven scientific articles addressing topics related to different primary and secondary raw materials. The articles offer advanced approaches to explore different aspects within mineral material life cycles (both primary and secondary): geological occurrences and extraction, processing, engineered mineral materials (cements, ceramics, alkali-activated materials, composites, and adsorbents), and their application and recycling. A summary of the contributions is listed below.

## 2. An Overview of the Published Articles

Clays were studied from two perspectives: natural glacial marine clays were tested for their use in fired bricks (contribution 1), and the structure of bentonite clays was studied to explore their application in refining recycled vegetable oil (contribution 2). Fine-grained glaciogene marine sediments are widespread in the northern hemisphere and can be utilized to produce local construction materials [33]. In contribution 1 [34], clay such as this from Greenland (pure or with 10 mass % (ma%) crushed granite residues or 10 ma% waste chamotte) was tested for the pilot production of fired bricks. The bricks were fired at temperatures between 1030 and 1080 °C. Temperatures from 1030 to 1055 °C proved to be sufficient, as higher firing temperatures did not improve the product’s technical properties. Granite reduced the mechanical properties of the bricks, while chammote had no effect on their properties. Such glacial marine clay proved to be suitable for the production of high-quality bricks. Additionally, bentonite clays are significant in the recycling of vegetable oils (contribution 2 [35]), which can become contaminated with metals and organic molecules from cooking equipment, as well as through the Maillard reaction [36] and food leaching [37]. Hydrophilic and hydrophobic commercial bentonites and modified (ground) bentonites from waste sources were investigated using ^29^Si solid-state NMR, which revealed differences in the structures of hydrophilic and hydrophobic bentonites. The differences between commercial bentonite and modified bentonite were revealed by ^27^Al solid-state NMR spectra. The original hydrophobic bentonite was more efficient at trapping ketones than the ground bentonite. The morphology and chemical structure of bentonite proved to be a crucial parameter influencing the efficiency of vegetable oil refining.

Clinoptilolite-rich tuffs are environmentally friendly materials with a variety of potential applications (contribution 3 [38]). They are primarily composed of the natural zeolite mineral clinoptilolite, which forms in environments characterized by volcanic activity and hydrothermal processes. Its crystal lattice has an open and easily accessible structure, which makes it suitable for molecular sieves and ion exchange, ion adsorption, and ion separation [39]. The capacity for ion exchange varies depending on the Si/Al ratio and the type of extra-structural cations. Tuffs with a high clinoptilolite content are suitable for the removal of various inorganic and organic pollutants from wastewater. Clinoptilolite is stable both thermally and chemically, making it suitable for the production of catalytically active oxides. Additionally, it enhances the nutrient properties of soil and plays an important role in the biotechnological treatment of activated sludge and wastewater.

Various Al-containing cement binders are gaining importance globally due to their lower ecological footprint compared to ordinary OPC binders. However, one significant challenge is ensuring that there are enough Al-rich industrial residues for large-scale production of such clinkers, in alignment with the principles of the circular economy and the Green Deal. Al-containing industrial residues were collected in the ESEE region, which included red mud from alumina production, various slags from the steel industry, fly and bottom ash from thermal power plants, fly and bottom ash from paper mills, and other industrial residues (contribution 4 [40]). The materials were characterized in terms of their physical, chemical, mineralogical, and radiological properties. Although the investigated SRMs were not suitable for the recovery of base metals or REEs, they were evaluated from an environmental and radiological perspective. The evaluation revealed that these materials possess suitable properties for potential use in conventional building products, as well as for products that support the green transition, such as alkali-activated materials, aerogels, zeolites, etc. The amount of SRMs that can be incorporated depends on the desired chemical and mineralogical composition of the product. This quantity can be increased through appropriate pre-treatment of the SRMs. In contrast to the investigated Al-containing SRMs, ashes from sewage sludge (SS), municipal solid waste (MSW), or wood biomass (WB) have shown great potential for the recovery of metals and phosphorus (P) (contribution 5 [41]). An environmentally friendly alternative to the usual wet-chemical extraction processes is extraction based on electrochemical technologies. These methods allow for the utilization of electricity from renewable sources and enable extraction during periods of surplus grid energy. In addition, the pre-treatment of such ash by removing heavy metals makes these materials even more suitable for various applications (a substitute material for cement, filler or fine aggregate components in mortar and concrete and a substitute material for clay in bricks, precursor materials for alkali activation, etc.). Particular attention has been given to WB ashes, which have the potential to act as a CO_2_ sink through mineral carbonation.

The stabilization of natural fine-grained soils using cement and sawdust ash (SDA) as a clayey linear material (contribution 6 [42]), as well as eco-cements with maraboo weed biomass ash (MA) (contribution 7 [43]), exemplifies a circular economy approach. SDA is a low-cost, environmentally friendly waste material with pozzolanic properties that can improve the mechanical properties of cementitious materials [44]. It has been found that SDA can replace soil by up to 10% and cement by up to 9% without losing performance, while optimal mechanical properties of the composite were achieved for a mixture containing 6% cement and 6% SDA based on the dry weight of the soil. The main hydration products were calcium silicate hydrate gels (C-S-H) and double-layer hydroxide compounds, which were recoverable over a period of one year. However, long-term monitoring of the performance of the stabilized soil is required to evaluate its durability under actual environmental conditions. The replacement of ordinary Portland cement (OPC) with 10 or 20 ma% MA in the binary cements CEM II-A (6–20%), fired at 600 °C, was studied in contribution 7 [43]. The mortars were mixed at a cement-to-sand ratio of 1:3 and a water-to-cement ratio of 0.5. The MA showed medium–low pozzolanic activity, which was attributed to the low acid oxide content and high loss on ignition. Both the 10 and 20 ma% MA-blended cements met the prescribed chemical, physical, and mechanical requirements for MAs to be classified as supplementary cementitious materials. The alkaline aluminosilicate binders of the Na_2_O(K_2_O)-Al_2_O_3_-SiO_2_-H_2_O system with different oxide ratios (Na_2_O(K_2_O)/Al_2_O_3_ and SiO_2_/Al_2_O_3_) are presented in contribution 8 [45]. Both binders can be regarded as advanced, environmentally friendly mineral binders. The developed binder is based on the alkali activation of metakaolin and kaolin. The method was validated through pilot studies and small-scale industrial production, along with their corresponding applications. Binders with specific oxide ratios (Na,K)_2_O/Al_2_O_3_ = 1, SiO_2_/Al_2_O_3_ = 2 to 7, and H_2_O/Al_2_O_3_ = 10 to 15) resulted in the formation of zeolite-like products. The hardening process was attributed to the formation of aluminosilicate hydrates via the following stages: amorphous, sub-microcrystalline, and crystalline. At a ratio H_2_O/Al_2_O_3_ > 10, the sub-microcrystalline structure became barely recognizable. This resulted in the nucleation of large crystals in the amorphous phase, which slowed down the hardening and crystallization process, leading to a decline in the product’s properties. This study provides an innovative approach for the development of an alkaline aluminosilicate binder that can be used in various thermal insulation and fire protection applications, including glues and adhesives.

Geotechnical aspects of mineral materials are investigated in contributions 9 and 10. Contribution 9 [46] presents a new analytical model for predicting the mechanical behavior of brittle sandstones with a dry density of 2.153 to 2.659 g/cm^3^ under triaxial compression. The model is based on the wing crack model developed by Ashby and Hallam [47]. It aims to determine the normalized critical crack length by which the fracture strength can be estimated based on fracture mechanics applied to wing cracks emanating from the tips of pre-existing cracks. The model is mainly applicable to rocks with Ψ-angles < 30°, which corresponds to tensile failure. Contribution 10 [48] investigates the accuracy of bonded block models (BBMs) in predicting the analogous mechanical behavior of large-scale rock formations (rockmass), which are difficult to test directly in the laboratory due to the required sample scale. The behavior of rock masses at the field scale is usually simulated by Synthetic Rockmass Modeling (SRM) [37]. The aim of this study was to validate the SRM method on Blanco Mera granite using existing results from laboratory tests. A calibrated Discrete Element Model (DEM) of the intact rock and a Discrete Fracture Network (DFN) were created. The study included an elastic constitutive model and an inelastic constitutive model of the blocks. Both BBMs successfully predicted the pre-peak properties and peak strength of the joined models. The models predicted the strength, dilatation coefficient, and microfracture behavior of the joined laboratory specimens.

This issue contains also study about new advances in mineralogy. New minerals were found in corundum xenocrysts from the pyroclastic ejecta of small Cretaceous basaltic volcanoes in the area of Mt. Carmel, Israel [49], which are formed under reduction conditions in the Earth’s upper mantle. The following new minerals have been described in this area since 2021: griffinite (Al_2_TiO_5_), magnéliite (Ti^3+^_2_Ti^4+^_2_O_7_), ziroite (ZrO_2_), sassite (Ti^3+^_2_Ti^4+^O_5_), mizraite-(Ce) (Ce(Al_11_Mg)O_19_), toledoite (TiFeSi), and yeite (TiSi). Five other new high-temperature oxide or alloy minerals are presented, namely magnéliite (Ti^3+^_2_Ti^4+^_2_O_7_), ziroite (ZrO_2_), sassite (Ti^3+^_2_Ti^4+^O_5_), mizraite-(Ce) (Ce(Al_11_Mg)O_19_), and yeite (TiSi), as presented in contribution 11 [50]. The minerals were nanoscale in size, limiting the determination of all physical properties. This study provides a description of their chemical composition and the crystal structures of synthetic analogs.

## 3. Conclusions

This compilation presents recent advancements in the science of rock and mineral raw materials. It focuses on studies related to clays, new forms of cement and their applications, new advancements in mineralogy and metal recovery, and the mechanical properties of rocks. Since cement production has a significant environmental impact worldwide, it is crucial to study and develop new eco-friendly mineral binders. These advancements have the potential to drive the construction sector toward achieving climate neutrality and goals related to the circular economy.

Clay, due to its layered molecular structure, exhibits various interesting properties. When heated, clays can function as a binder and can also act as adsorbents or a molecular sieve. These properties are important for the construction, production of ceramics, waste treatment, and other applications (for example, as lubricants). Investigating such properties of clay offers the potential to discover their various other applications, including uses in the electrotechnical industry (as insulators or even as superconductors).

Geologically conditioned hazards, such as rockfalls, pose a risk to infrastructure, and identifying these risks can save both money and lives. Investigating the mechanical properties of rock mass is of vital importance for ensuring the safety and quality of infrastructure and buildings, especially in mountainous areas.

Finally, new advancements in mineralogy are opening up new applications across various sectors, including, e.g., optics.

A collection of advancements in the above-mentioned field is presented in the edited issue “Advances in Rock and Mineral Materials”. The editors hope this knowledge will improve the everyday lives of humankind.

## References

[1] Ghafoor S., Shooshtarian S., Udawatta N., Gurmu A., Karunasena G., Maqsood T. (2024). Cost factors affecting the utilisation of secondary materials in the construction sector: A systematic literature review. Resour. Conserv. Recycl. Adv..

[2] Remeikienė R., Gasparėnienė L., Matulienė S., Szarucki M. (2024). Secondary Raw Materials in the Circular Economy: A Multi-Perspective Study.

[3] Lundaev V., Solomon A.A., Le T., Lohrmann A., Breyer C. (2023). Review of critical materials for the energy transition, an analysis of global resources and production databases and the state of material circularity. Miner. Eng..

[4] Božič M., Žibret L., Kvočka D., Pranjić A.M., Gregorc B., Ducman V. (2023). Drava river sediment in clay brick production: Characterization, properties, and environmental performance. J. Build. Eng..

[5] Ottosen L.M., Bertelsen I.M.G., Jensen P.E., Kirkelund G.M. (2020). Sewage sludge ash as resource for phosphorous and material for clay brick manufacturing. Constr. Build. Mater..

[6] Czerwinski F. (2022). Critical Minerals for Zero-Emission Transportation. Materials.

[7] Kriven W.M., Leonelli C., Provis J.L., Boccaccini A.R., Attwell C., Ducman V.S., Ferone C., Rossignol S., Luukkonen T., Van Deventer J.S.J. (2024). Why geopolymers and alkali-activated materials are key components of a sustainable world: A perspective contribution. J. Am. Ceram. Soc..

[8] González I., Galán E., Miras A., Vázquez M.A. (2011). CO2 emissions derived from raw materials used in brick factories. Applications to Andalusia (Southern Spain). Appl. Clay Sci..

[9] Brumaud C., Du Y., Ardant D., Habert G. (2024). Earth, the new liquid stone: Development and perspectives. Mater. Today Commun..

[10] Lovec V., Jovanovic-Popovic M., Zivkovic B. (2018). The thermal behavior of rammed earth wall in traditional house in Vojvodina: Thermal mass as a key element for thermal comfort. Therm. Sci..

[11] Oti J.E., Kinuthia J.M. (2012). Stabilised unfired clay bricks for environmental and sustainable use. Appl. Clay Sci..

[12] Muheise-Araalia D., Pavia S. (2021). Properties of unfired, illitic-clay bricks for sustainable construction. Constr. Build. Mater..

[13] Mannu A., Di Pietro M.E., Petretto G.L., Taleb Z., Serouri A., Taleb S., Sacchetti A., Mele A. (2023). Recycling of used vegetable oils by powder adsorption. Waste Manag. Res..

[14] Xie S., Huang L., Su C., Yan J., Chen Z., Li M., Du M., Zhang H. (2024). Application of clay minerals as adsorbents for removing heavy metals from the environment. Green Smart Min. Eng..

[15] Colella C., Gennaro M.D., Aiello R. (2001). Use of Zeolitic Tuff in the Building Industry. Rev. Miner. Geochem..

[16] Margeta K., Stefanović Š.C., Kaučič V., Logar N.Z. (2015). The potential of clinoptilolite-rich tuffs from Croatia and Serbia for the reduction of toxic concentrations of cations and anions in aqueous solutions. Appl. Clay Sci..

[17] Gartner E., Sui T. (2018). Alternative cement clinkers. Cem. Concr. Res..

[18] Žibret L., Ipavec A., Dolenec S. (2022). Microstructural characteristics of belite–sulfoaluminate cement clinkers with bottom ash. Constr. Build. Mater..

[19] Bullerjahn F., Schmitt D., Ben Haha M. (2014). Effect of raw mix design and of clinkering process on the formation and mineralogical composition of (ternesite) belite calcium sulphoaluminate ferrite clinker. Cem. Concr. Res..

[20] Žibret G., Teran K., Žibret L., Šter K., Dolenec S. (2021). Building of the Al-containing Secondary Raw Materials Registry for the Production of Low CO_2_ Mineral Binders in South-Eastern European Region. Sustainability.

[21] Carević I., Serdar M., Štirmer N., Ukrainczyk N. (2019). Preliminary screening of wood biomass ashes for partial resources replacements in cementitious materials. J. Clean. Prod..

[22] Bernal S.A., Rodríguez E.D., Kirchheim A.P., Provis J.L. (2016). Management and valorisation of wastes through use in producing alkali-activated cement materials: Wastes producing alkali-activated cement materials. J. Chem. Technol. Biotechnol..

[23] Adesanya E., Dabbebi R., Rößler C., Pavlin M., Li Z., Luukkonen T., Yliniemi J., Illikainen M. (2024). Analysis of alkali-activated mineral wool-slag binders: Evaluating the differences between one-part and two-part variations. J. Mater. Cycles Waste Manag..

[24] Bílek V., Novotný R., Koplík J., Kadlec M., Kalina L. (2023). Philosophy of rational mixture proportioning of alkali-activated materials validated by the hydration kinetics of alkali-activated slag and its microstructure. Cem. Concr. Res..

[25] Kryvenko P., Rudenko I., Kovalchuk O., Gelevera O., Konstantynovskyi O. (2023). Influence of Dosage and Modulus on Soluble Sodium Silicate for Early Strength Development of Alkali-Activated Slag Cements. Minerals.

[26] Chen B., Ye G. (2025). Enhancing the reaction of municipal solid waste incineration (MSWI) bottom ash in blast furnace slag-based alkali-activated blends: A novel strategy and underlying mechanism. Cem. Concr. Compos..

[27] Chen B., Perumal P., Liu C., Chen Y., Chang C., Pavlin M., Kvočka D., Ducman V., Luukkonen T., Illikainen M. (2025). Municipal solid waste incineration (MSWI) bottom ash-blended cementitious materials: Performance, challenges, and potential solutions. Crit. Rev. Environ. Sci. Technol..

[28] Lima A.T., Ottosen L. (2021). Recovering rare earth elements from contaminated soils: Critical overview of current remediation technologies. Chemosphere.

[29] Ottosen L.M., Kirkelund G.M., Jensen P.E. (2013). Extracting phosphorous from incinerated sewage sludge ash rich in iron or aluminum. Chemosphere.

[30] Wen Y., Hu L., Boxleiter A., Li D., Tang Y. (2024). Rare Earth Elements Recovery and Waste Management of Municipal Solid Waste Incineration Ash. ACS Sustain. Resour. Manag..

[31] Wang N., Bai Y., Guo Z., Fan Y., Meng F. (2024). Synergies between the circular economy and carbon emission reduction. Sci. Total Environ..

[32] Scrivener K.L., John V.M., Gartner E.M. (2018). Eco-efficient cements: Potential economically viable solutions for a low-CO2 cement-based materials industry. Cem. Concr. Res..

[33] Belmonte L.J., Ottosen L.M., Kirkelund G.M., Jensen P.E., Vestbø A.P. (2018). Screening of heavy metal containing waste types for use as raw material in Arctic clay-based bricks. Environ. Sci. Pollut. Res..

[34] Belmonte L.J., Ottosen L.M., Kirkelund G.M. (2024). Use of a Glaciogene Marine Clay (Ilulissat, Greenland) in a Pilot Production of Red Bricks. Materials.

[35] Mannu A., Castia S., Petretto G., Garroni S., Castiglione F., Mele A. (2025). Exploring the Structure–Activity Relationship of Bentonites for Enhanced Refinement of Recycled Vegetable Oil. Materials.

[36] Shi B., Guo X., Liu H., Jiang K., Liu L., Yan N., Farag M.A., Liu L. (2024). Dissecting Maillard reaction production in fried foods: Formation mechanisms, sensory characteristic attribution, control strategy, and gut homeostasis regulation. Food Chem..

[37] Kuek S.L., Ahmad Tarmizi A.H., Abd Razak R.A., Jinap S., Norliza S., Sanny M. (2020). Contribution of lipid towards acrylamide formation during intermittent frying of French fries. Food Control.

[38] Pavlović J., Hrenović J., Povrenović D., Rajić N. (2024). Advances in the Applications of Clinoptilolite-Rich Tuffs. Materials.

[39] Dziedzicka A., Sulikowski B., Ruggiero-Mikołajczyk M. (2016). Catalytic and physicochemical properties of modified natural clinoptilolite. Catal. Today.

[40] Fidanchevski E., Šter K., Mrak M., Rajacic M., Koszo B.D., Ipavec A., Teran K., Žibret G., Jovanov V., Aluloska N.S. (2024). Characterization of Al-Containing Industrial Residues in the ESEE Region Supporting Circular Economy and the EU Green Deal. Materials.

[41] Tominc S., Ducman V., Wisniewski W., Luukkonen T., Kirkelund G.M., Ottosen L.M. (2023). Recovery of Phosphorus and Metals from the Ash of Sewage Sludge, Municipal Solid Waste, or Wood Biomass: A Review and Proposals for Further Use. Materials.

[42] Iliyas S., Idris A., Umar I.H., Lin H., Muhammad A., Xie L. (2024). Experiment and Analysis of Variance for Stabilizing Fine-Grained Soils with Cement and Sawdust Ash as Liner Materials. Materials.

[43] Frías M., Moreno De Los Reyes A.M., Villar-Cociña E., García R., Vigil De La Villa R., Vasić M.V. (2024). New Eco-Cements Made with Marabou Weed Biomass Ash. Materials.

[44] Yagüe S., González Gaya C., Rosales Prieto V., Sánchez Lite A. (2020). Sustainable Ecocements: Chemical and Morphological Analysis of Granite Sawdust Waste as Pozzolan Material. Materials.

[45] Kryvenko P., Rudenko I., Konstantynovskyi O., Gelevera O. (2024). Design, Characterization, and Incorporation of the Alkaline Aluminosilicate Binder in Temperature-Insulating Composites. Materials.

[46] Alomari E., Ng K., Khatri L. (2024). An Expanded Wing Crack Model for Fracture and Mechanical Behavior of Sandstone Under Triaxial Compression. Materials.

[47] Ashby M.F., Hallam (Née Cooksley) S.D. (1986). The failure of brittle solids containing small cracks under compressive stress states. Acta Metall..

[48] West I., Walton G., Sinha S. (2023). Evaluating the Accuracy of Bonded Block Models for Prediction of Rockmass Analog Mechanical Behavior. Materials.

[49] Griffin W.L., Huang J.X., Thomassot E., Gain S.E.M., Toledo V., O’Reilly S.Y. (2018). Super-reducing conditions in ancient and modern volcanic systems: Sources and behaviour of carbon-rich fluids in the lithospheric mantle. Mineral. Petrol..

[50] Ma C., Cámara F., Bindi L., Toledo V., Griffin W.L. (2023). New Minerals from Inclusions in Corundum Xenocrysts from Mt. Carmel, Israel: Magnéliite, Ziroite, Sassite, Mizraite-(Ce) and Yeite. Materials.

